# Identification of a Possible Endocannabinoid-Mediated Mechanism of Action of Cetylated Fatty Acids

**DOI:** 10.3390/biom15030363

**Published:** 2025-03-02

**Authors:** Giulia Bononi, Carlotta Granchi, Tiziano Tuccinardi, Filippo Minutolo

**Affiliations:** 1 Department of Pharmacy, University of Pisa, Via Bonanno 6, 56126 Pisa, Italy; giulia.bononi@unipi.it (G.B.); carlotta.granchi@unipi.it (C.G.); tiziano.tuccinardi@unipi.it (T.T.); 2 Center for Instrument Sharing of the University of Pisa (CISUP), Lungarno Pacinotti 43, 56126 Pisa, Italy

**Keywords:** endocannabinoid system, cetylated fatty acids, monoacylglycerol lipase, MAGL inhibitors, analgesic agents, anti-inflammatory agents

## Abstract

Some musculoskeletal disorders, including osteoarthritis; arthrosis; post-traumatic injuries; and other inflammatory tendon, joint and muscular afflictions, still represent unmet medical needs. Cetylated fatty acids (CFAs) are key components of widely distributed over-the-counter products, especially for topical use, which are intended to reduce symptoms associated with these conditions. Nevertheless, the mechanism of action of CFAs’ analgesic and anti-inflammatory properties has not yet been clearly established. Endocannabinoids, such as 2-arachidonoylglycerol (2-AG) and anandamide (AEA), are known to produce analgesic and anti-inflammatory effects. These compounds undergo physiological inactivation operated by several enzymes, including monoacylglycerol lipase (MAGL). We herein demonstrate for the first time that the therapeutic effects of CFAs may be attributable, at least in part, to their MAGL inhibition activities, which induce a local increase in analgesic/anti-inflammatory endocannabinoids in close proximity to the site of administration. These findings pave the way for the development of new potent local analgesic agents, whose action is based on an indirect cannabinoid effect.

## 1. Introduction

Musculoskeletal disorders, including osteoarthritis, tendinitis, and various forms of soft tissue inflammation, represent a significant burden on global health. These conditions often lead to chronic pain, reduced mobility, and a diminished quality of life. Conventional treatments for musculoskeletal pathologies typically involve the use of nonsteroidal anti-inflammatory drugs (NSAIDs), corticosteroids, and physical therapy. However, these approaches can be associated with a range of side effects and limitations, prompting the need for alternative therapeutic strategies [1].

**Figure 1:**
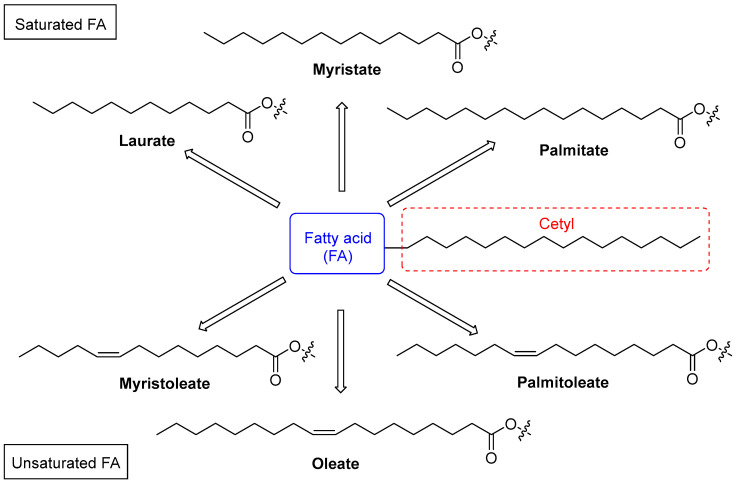
Some representative examples of naturally occurring CFAs, both saturated and unsaturated, present in most topical preparations for the treatment of musculoskeletal disorders. The common cetyl portion is highlighted in the red dashed square.

Cetylated fatty acids (CFAs) have emerged as a promising class of compounds in the management of musculoskeletal disorders, demonstrating anti-inflammatory and pain-relieving properties. The most renowned CFA is cetyl myristoleate, also known as CMO (Figure 1), which was first identified for its potential in treating arthritis [2]. From a molecular perspective, CFAs are naturally occurring lipophilic esters derived from plant and/or animal sources, consisting of fatty acids esterified with cetyl alcohol (possessing a linear saturated C16 alkyl chain). In addition to the aforementioned CMO, this series of compounds generally includes other analogues such as cetyl myristate, cetyl palmitoleate, cetyl palmitate, cetyl oleate, and cetyl laurate (Figure 1) [3,4].

CFAs are commonly administered topically due to their rapid absorption via passive permeation through cell membranes, facilitated by their lipid nature [5]. They are primarily used in therapy for their anti-inflammatory and analgesic properties, particularly in the management of joint pain, inflammation, and muscle recovery, as well as for improving joint mobility in conditions such as osteoarthritis and rheumatoid arthritis [3,6,7,8,9]. Recent studies have demonstrated the efficacy of topical CFAs in treating symptoms associated with athletic pubalgia in professional roller hockey players [10], shoulder tendinopathies [11], knee osteoarthritis [12], and chronic neck pain [4,13]. A comparative study of their effect on the production of inflammatory cytokine IL-6 in mouse macrophage cells demonstrated that treatment with 0.7 mg/mL of CFAs produced a higher degree of reduction in IL-6 levels than 1 mM ibuprofen, 0.5 mM prednisone, or 0.5 mM piroxicam, demonstrating that CFAs may be considered valid alternatives to NSAIDs and steroids to counteract this type of inflammation without significant side effects [3].

CFAs are believed to exert their therapeutic effects through various mechanisms, including modulation of immune responses, lubrication of joints, and inhibition of inflammatory mediators. Nevertheless, while numerous products containing these compounds are currently available and widely used, their exact mechanism of action still remains incompletely understood. A mechanism proposed in 1994 suggested that CFAs exert their effects by stabilizing cell membranes and protecting synovial tissues [14]. These actions help maintain normal joint flexibility and mobility, alleviate pain, and enhance the production of joint fluid, thereby supporting proper lubrication [9,11,14]. However, more specific molecular targets that explain their mechanism of action have not yet been identified.

Over the past few years, the endocannabinoid system (ECS) has emerged as a critical regulator of numerous physiological and pathological processes, including pain modulation, inflammation, neuroprotection, and metabolic homeostasis [15,16], offering potential therapeutic targets for managing several conditions. The ECS is a complex signalling network where the enzymes fatty-acid amide hydrolase (FAAH) and monoacylglycerol lipase (MAGL) play critical roles in terminating endocannabinoid signalling. FAAH catalyses the hydrolysis of amide-type endocannabinoids, such as anandamide and other *N*-acylethanolamides. On the other hand, MAGL promotes the hydrolysis of ester-type substrates, such as 2-arachidonoylglycerol (2-AG), the most abundant endocannabinoid ligand, into arachidonic acid and glycerol. This activity not only regulates 2-AG levels but also contributes to the biosynthesis of pro-inflammatory prostaglandins via arachidonic acid metabolism [17,18]. Emerging evidence has highlighted the therapeutic potential of MAGL inhibition in a variety of disorders, particularly those involving chronic pain and neuroinflammation [19,20,21,22,23]. The inhibition of MAGL elevates endogenous 2-AG levels, leading to enhanced activation of cannabinoid receptors CB1 and CB2, which are known to mediate analgesic and anti-inflammatory effects [24]. Moreover, by limiting the availability of arachidonic acid for cyclooxygenase pathways, MAGL inhibitors may simultaneously reduce pro-inflammatory eicosanoid production, providing a dual therapeutic effect [18,25].

Numerous academic institutions and pharmaceutical companies have worked on the development of MAGL inhibitors, employing both reversible and irreversible mechanisms of action [26,27,28,29,30,31,32,33]. While irreversible MAGL inhibitors often demonstrate strong inhibitory potency, their primary limitation lies in the side effects observed during in vivo studies [27,34]. Prolonged elevation of 2-AG levels resulting from their use can lead to CB1 receptor desensitization. Additionally, studies on mice treated with irreversible MAGL inhibitors or genetically lacking MAGL have revealed disrupted CB1-mediated synaptic plasticity, cross-tolerance to external CB1 agonists, and signs of physical dependence [27,34].

Recent studies have demonstrated that reversible MAGL inhibitors offer a promising strategy for nociceptive pain management with reduced risks of side effects associated with irreversible inhibitors, such as the compensatory upregulation of MAGL or off-target toxicities [21,24,25,35,36,37,38,39,40,41]. The development of potent, selective, and reversible MAGL inhibitors has therefore become an area of active research, with the goal of harnessing their nociceptive properties for clinical applications.

The aim of this article is to support the involvement of the ECS in the mechanism of action of CFAs and to discuss potential challenges and future directions for research in this field.

Considering the molecular structures of CFAs and their partial similarities to portions of some ester-based endocannabinoids (for example, 2-AG, virodhamine, etc.), we were intrigued by the possibility that they could act as false substrates in the enzyme that is primarily responsible for their degradation, MAGL, and that they could act as inhibitors of this enzyme, thus producing their final effects through an ECS-mediated mechanism. Therefore, we planned to obtain pure representative CFAs and to test the activity of each of them on the isolated human MAGL (*h*MAGL) enzyme, with the purpose of determining whether the reported local analgesic and anti-inflammatory activity of these compounds could be in part attributed to MAGL inhibition. At the same time, the aim of our work also included the identification of which of these CFAs displays the highest MAGL inhibition potency.

## 2. Materials and Methods

### 2.1. Chemical Synthesis: Materials and General Procedures

All chemicals and solvents were utilized as obtained from commercial sources without further purification. Purifications by means of chromatographic techniques were achieved on silica gel columns by flash chromatography (Kieselgel 40, 0.040−0.063 mm; Merck, Darmstadt, Germany). The reaction outcomes were monitored by thin layer chromatography (TLC) on Merck aluminium silica gel (60 F254) sheets and spots were detected under a UV lamp. Solvent removal was performed in vacuo (rotating evaporator). Na_2_SO_4_ was the drying agent utilized in all the procedures. Proton (^1^H) and carbon (^13^C) NMR spectra were obtained with a Bruker (Billerica, Massachussets) Avance III 400 MHz spectrometer using the indicated deuterated solvents. The values of the chemical shifts are reported as parts per million (ppm) (δ relative to residual solvent peak for ^1^H and ^13^C). ^1^H-NMR spectra are described in the following order: multiplicity and number of protons derived from integrations of the peak areas. Standard abbreviations were used to indicate the signal multiplicity: t = triplet and m = multiplet. The ESI-MS (Electrospray Mass Spectrometry) spectra were recorded by direct injection at a 5 μL min^−1^ flow rate in an Orbitrap high-resolution mass spectrometer (Thermo, San Jose, CA, USA), provided with a HESI (Heated Electrospray Ionization) source under the following working conditions: sheath gas set at 24, auxiliary gas set at 5 (arbitrary units), positive polarity, spray voltage of 3.4 kV, capillary temperature of 290 °C, and S-lens RF level 50. Xcalibur 4.2 software (Thermo) was used for acquisition and analysis. For spectra acquisition, a nominal resolution (at *m*/*z* 200) of 140,000 was employed. The reaction yields referred to the amounts of isolated and purified products obtained in non-optimized procedures.

#### 2.1.1. General Procedure for the Formation of Final Compounds **3b**,**d**,**e**

In a two-neck round-bottom flask, flame-dried, and under an Argon atmosphere, commercially available decanoic acid **2b**, tetradecanoic acid **2d**, or palmitic acid **2e** (1.0 equiv. 0.82 mmol) was dissolved in anhydrous acetonitrile (2.5 mL). 1,1′-Carbonyldiimidazole (CDI) (1.0 equiv., 0.82 mmol) was then added, and the mixture was stirred and heated at 50 °C for 1 h. Subsequently, 1-hexadecanol **1** (1.00 equiv., 0.825 mmol) was added, and the mixture was heated at 65 °C for 3 h. After confirming the complete consumption of the starting material, water was added to the reaction mixture. The reaction mixture was extracted with ethyl acetate (3x), and the organic phase was washed with brine (1x), dried over Na_2_SO_4_, filtered, and concentrated under reduced pressure. The crude product was purified by silica gel column chromatography. Elution with *n*-hexane or *n*-hexane/ethyl acetate 95:5 afforded the desired esters **3b**,**d**,**e**.

*Hexadecyl decanoate* **3b**. White solid; 21% yield from hexadecan-1-ol **1** and decanoic acid **2b**. ^1^H-NMR (CDCl_3_) δ (ppm): 0.87 (t, 6H, *J* = 6.8 Hz), 1.20–1.36 (m, 38H), 1.56–1.66 (m, 4H), 2.28 (t, 2H, *J* = 7.6 Hz), 4.05 (t, 2H, *J* = 6.7 Hz). ^13^C-NMR (CDCl_3_) δ (ppm): 14.23, 14.24, 22.80, 22.82, 25.18, 26.08, 28.81, 29.31, 29.40, 29.42, 29.50, 29.57, 29.67, 29.71, 29.79, 29.81, 29.83, 31.04, 32.00, 32.06, 34.57, 64.54, 174.15. HRMS: *m*/*z* for C_26_H_53_O_2_ [M + H]^+^ calculated: 397.40456, found: 397.40329.

*Hexadecyl tetradecanoate* **3d**. White solid; 52% yield from hexadecan-1-ol **1** and tetradecanoic acid **2d**. ^1^H-NMR (CDCl_3_) δ (ppm): 0.88 (t, 6H, *J* = 6.9 Hz), 1.20–1.37 (m, 46H), 1.56–1.66 (m, 4H), 2.28 (t, 2H, *J* = 7.6 Hz), 4.05 (t, 2H, *J* = 6.7 Hz). ^13^C-NMR (CDCl_3_) δ (ppm): 14.26 (2C), 22.84, 25.19, 26.09, 28.81, 29.32, 29.43, 29.51, 29.63, 29.68, 29.73, 29.76, 29.81, 29.84, 32.08, 34.58, 64.55, 174.17. HRMS: *m*/*z* for C_30_H_60_O_2_Na [M + Na]^+^ calculated: 475.44910, found: 475.44855.

*Hexadecyl pentadecanoate* **3e**. White solid; 7% yield from hexadecan-1-ol **1** and palmitic acid **2e**. ^1^H-NMR (CDCl_3_) δ (ppm): 0.88 (t, 6H, *J* = 6.8 Hz), 1.19–1.37 (m, 50H), 1.56–1.66 (m, 4H), 2.29 (t, 2H, *J* = 7.5 Hz), 4.05 (t, 2H, *J* = 6.7 Hz). ^13^C-NMR (CDCl_3_) δ (ppm): 14.28 (2C), 22.86 (2C), 25.21, 26.11, 28.82, 29.33, 29.43, 29.44, 29.53, 29.65, 29.70, 29.75, 29.77, 29.82, 29.86, 31.75, 32.09, 34.60, 64.57, 174.20. HRMS: *m*/*z* for C_32_H_64_O_2_Na [M + Na]^+^ calculated: 503.48040, found: 503.47985.

#### 2.1.2. General Procedure for the Formation of Final Compounds **3a**,**c**,**f**–**i**

In a two-neck round-bottom flask, flame-dried, and under an Argon atmosphere, commercially available hexanoic acid **2a**, dodecanoic acid **2c**, stearic acid **2f**, *(Z)*-tetradec-9-enoic acid **2g**, *(Z)*-hexadec-9-enoic acid **2h**, or oleic acid **2i** (1.0 equiv., 0.37 mmol) was dissolved in anhydrous dimethylformamide (1.8 mL) under stirring. Subsequently, 1-ethyl-3-(3-dimethylaminopropyl)carbodiimide (EDCI) (1.1 equiv., 0.41 mmol) and hydroxybenzotriazole (HOBt) (1.1 equiv., 0.41 mmol) were added. After 15 min, 1-hexadecanol **1** (1.1 equiv., 0.41 mmol) and diisopropylethylamine (DIPEA, 1.1 equiv., 0.41 mmol) were added. The mixture was stirred overnight at room temperature. Upon confirming the complete consumption of the starting material, the reaction mixture was extracted with ethyl acetate (3x), and the organic phase was washed with brine (2x), dried over Na_2_SO_4_, filtered, and concentrated under reduced pressure. The crude product was purified by silica gel column chromatography using a *n*-hexane/ethyl acetate mixture 95:5 as the eluent, yielding the desired esters **3a**,**c**,**f**–**i**.

*Hexadecyl hexanoate* **3a**. Colourless oil; 39% yield from hexadecan-1-ol **1** and hexanoic acid **2a**. ^1^H-NMR (CDCl_3_) δ (ppm): 0.88 (q, 6H, *J* = 6.7 Hz), 1.22–1.37 (m, 30H), 1.57–1.67 (m, 4H), 2.29 (t, 2H, *J* = 7.6 Hz), 4.05 (t, 2H, *J* = 6.8 Hz). ^13^C-NMR (CDCl_3_) δ (ppm): 14.06, 14.26, 22.47, 22.84, 24.86, 26.08, 28.79, 29.40, 29.51, 29.67, 29.71, 29.79, 29.80, 29.81, 29.84, 31.47, 32.07, 34.52, 64.55, 174.17. HRMS: *m*/*z* for C_22_H_45_O_2_ [M + H]^+^ calculated: 341.34196, found: 341.34141.

*Hexadecyl dodecanoate* **3c**. White solid; 35% yield from hexadecan-1-ol **1** and dodecanoic acid **2c**. ^1^H-NMR (CDCl_3_) δ (ppm): 0.88 (t, 6H, *J* = 6.8 Hz), 1.20–1.37 (m, 42H), 1.56–1.66 (m, 4H), 2.28 (t, 2H, *J* = 7.6 Hz), 4.05 (t, 2H, *J* = 6.7 Hz). ^13^C-NMR (CDCl_3_) δ (ppm): 14.26, 22.84, 25.19, 26.10, 28.82, 29.32, 29.41, 29.43, 29.49, 29.51, 29.62, 29.68, 29.73, 29.76, 29.80, 29.83, 29.85, 32.07, 32.08, 34.58, 64.55, 174.17. HRMS: *m*/*z* for C_28_H_60_O_2_N [M + NH_4_]^+^ calculated: 442.46241, found: 442.46186.

*Hexadecyl stearate* **3f**. White solid; 63% yield from hexadecan-1-ol **1** and stearic acid **2f**. ^1^H-NMR (CDCl_3_) δ (ppm): 0.88 (t, 6H, *J* = 6.8 Hz), 1.19–1.37 (m, 54H), 1.56–1.66 (m, 4H), 2.28 (t, 2H, *J* = 7.5 Hz), 4.05 (t, 2H, *J* = 6.7 Hz). ^13^C-NMR (CDCl_3_) δ (ppm): 14.23, 22.81, 25.15, 26.06, 28.77, 29.28, 29.37, 29.39, 29.41, 29.48, 29.59, 29.64, 29.70, 29.72, 29.77, 29.81, 32.04, 34.54, 64.52, 174.15. HRMS: *m*/*z* for C_34_H_68_O_2_Na [M + Na]^+^ calculated: 531.51170, found: 531.51115.

*Hexadecyl (Z)-tetradec-9-enoate* **3g**. Colourless oil; 49% yield from hexadecan-1-ol **1** and *(Z)*-tetradec-9-enoic acid **2g**. ^1^H-NMR (CDCl_3_) δ (ppm): 0.85–0.92 (m, 6H), 1.20–1.37 (m, 38H), 1.56–1.66 (m, 4H), 1.98–2.05 (m, 4H), 2.29 (t, 2H, *J* = 7.5 Hz), 4.05 (t, 2H, *J* = 6.7 Hz), 5.29–5.39 (m, 2H). ^13^C-NMR (CDCl_3_) δ (ppm): 14.14, 14.26, 22.49, 22.84, 25.17, 26.09, 27.07, 27.30, 28.81, 29.25, 29.28, 29.31, 29.41, 29.51, 29.68, 29.73, 29.81, 29.84, 32.08, 32.11, 34.56, 64.56, 129.92, 130.08, 174.15. HRMS: *m*/*z* for C_30_H_59_O_2_ [M + H]^+^ calculated: 451.45151, found: 451.45096.

*Hexadecyl (Z)-hexadec-9-enoate* **3h**. Colourless oil; 53% yield from hexadecan-1-ol **1** and *(Z)*-hexadec-9-enoic acid **2h**. ^1^H-NMR (CDCl_3_) δ (ppm): 0.88 (m, 6H), 1.20–1.38 (m, 42H), 1.56–1.66 (m, 4H), 1.97–2.05 (m, 4H), 2.29 (t, 2H, *J* = 7.5 Hz), 4.05 (t, 2H, *J* = 6.7 Hz), 5.29–5.39 (m, 2H). ^13^C-NMR (CDCl_3_) δ (ppm): 14.24, 14.26, 22.80, 22.83, 25.16, 26.08, 27.30, 27.36, 28.80, 29.13, 29.25, 29.27, 29.31, 29.40, 29.50, 29.67, 29.72, 29.79, 29.82, 29.83, 29.88, 31.93, 32.06, 34.55, 64.55, 129.90, 130.13, 174.14. HRMS: *m*/*z* for C_32_H_63_O_2_ [M + H]^+^ calculated: 479.48281, found: 479.48145.

*Hexadecyl oleate* **3i**. Colourless oil; 51% yield from hexadecan-1-ol **1** and oleic acid **2i**. ^1^H-NMR (CDCl_3_) δ (ppm): 0.88 (t, 6H, *J* = 6.8 Hz), 1.20–1.38 (m, 46H), 1.57–1.66 (m, 4H), 1.97–2.06 (m, 4H), 2.19 (t, 2H, *J* = 7.5 Hz), 4.05 (t, 2H, *J* = 6.7 Hz), 5.29–5.39 (m, 2H). ^13^C-NMR (CDCl_3_) δ (ppm): 14.26, 22.85, 25.18, 26.11, 27.33, 27.38, 28.83, 29.27, 29.30, 29.33, 29.42, 29.48, 29.52, 29.69, 29.74, 29.82, 29.86, 29.93, 32.07, 32.09, 34.57, 64.57, 129.91, 130.15, 174.14. HRMS: *m*/*z* for C_34_H_70_O_2_N [M + NH_4_]^+^ calculated: 524.54066, found: 524.54011.

### 2.2. MAGL Inhibition Assays

Human recombinant MAGL and 4-nitrophenyl acetate (4-NPA) were purchased from Cayman Chemical. *h*MAGL had a ≥95% purity rate estimated by SDSPAGE and was stored at −80 °C in order to preserve its stability, as recommended by the producer. IC_50_ values were determined using 96-well microtiter plates. The enzymatic reaction was conducted at room temperature in a final volume of 200 μL, prepared in 10 mM Tris buffer (pH 7.2) containing 1 mM EDTA and 0.1 mg/mL bovine serum albumin (BSA). A volume of 150 μL of 4-NPA at 133.3 μM was mixed with 10 μL of dimethylsulfoxide (DMSO) containing the desired concentration of the tested compound. The reaction was initiated by adding 40 μL of MAGL (11 ng per well), ensuring linearity over 30 min. The reference inhibitor JZL-184 [28] was used as a positive control. Initially, CFAs were tested at a single concentration of 50 μM, and the single-point percentage of inhibition was determined. Subsequently, the compound exhibiting the highest single-point percentage inhibition, compound **3b**, was further evaluated to determine its IC_50_ value. The final concentrations of the tested compound **3b** ranged from 50 µM to 0.78 µM. After 30 min, absorbance was measured at 405 nm using a Victor X3 Microplates Reader (PerkinElmer^®^, Shelton, Connecticut,). The control reactions included one without the test compound and another without both the compound and MAGL. The final data were obtained from duplicate measurements of three independent experiments. The spectrophotometric data were analysed and interpreted using the GraphPad Prism software, version 10.2.0. The data are presented as an average of 3 replicates (mean ± standard deviation), and a statistical analysis was performed using GraphPad Prism software. The IC_50_ value was calculated using the Sigmoidal dose–response fitting in GraphPad Prism software. To eliminate potential false positives, a blank analysis was carried out at each compound concentration. The final absorbance values were corrected by subtracting the absorbance measured in the absence of MAGL under identical conditions.

## 3. Results

### 3.1. Chemistry

**Figure 2:**
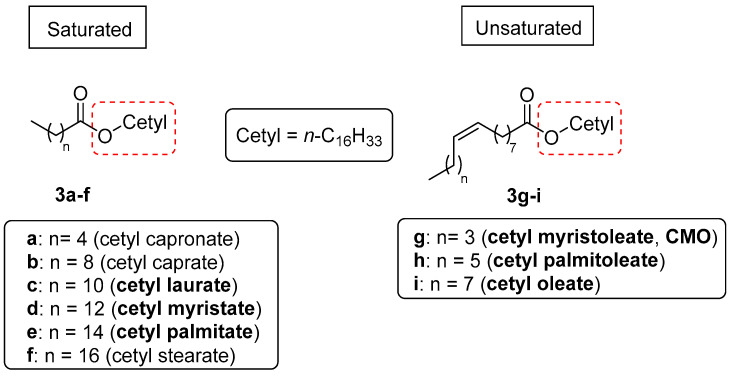
Structures of the synthesized CFAs **3a**–**i**. The cetylic portion is highlighted in the red dashed square. The names of CFAs commonly present in marketed topic formulations are highlighted in bold.

**Figure 3:**
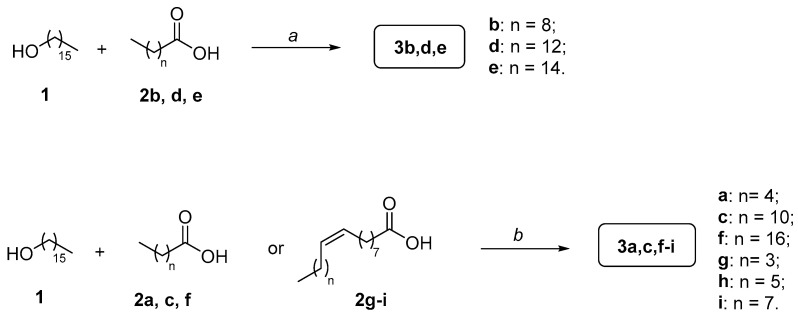
Synthesis of CFAs **3a**–**i**. Reagents and conditions: (*a*) CDI, anhydrous CH_3_CN, 80 °C, 3 h [7–52%]; (*b*) EDCI, HOBt, DIPEA, anhydrous DMF, rt, 12 h [35–63%].

We synthesized and purified nine CFAs **3a**–**i** (Figure 2). This series includes those that are commonly utilized in topical formulations, often as mixtures, containing both saturated (**3c**–**e**) and unsaturated fatty acid portions (**3g**–**i**), along with three additional analogues of the saturated series (**3a**,**b**,**f**), which were included to probe the effect of the length of the acyl chain on the MAGL inhibition properties of these compounds. The synthesis for obtaining compounds **3a**–**i** is outlined in Figure 3 and involves a rapid, single-step procedure based on the condensation of commercially available cetyl alcohol (**1**) with the respective commercially available carboxylic acids (**2a**–**i**), yielding the desired CFAs (**3a**–**i**) with variable yields.

Initially, commercially available cetyl alcohol (**1**) was condensed with the respective commercially available carboxylic acids **2b**,**d**,**e** in the presence of carbonyldiimidazole (CDI) as the coupling agent, using anhydrous acetonitrile (CH_3_CN) as the solvent and heating at 80 °C for three hours. This procedure yielded the first CFAs **3b**,**d**,**e** with moderate to good yields.

To enhance the reaction yields, the synthesis of the remaining CFAs was carried out under modified conditions. Based on literature precedents, commercially available cetyl alcohol **1** was condensed with the respective carboxylic acids **2a**,**c**,**f**–**i** in the presence of 1-ethyl-3-(3-dimethylaminopropyl)carbodiimide (EDCI) as the coupling agent, hydroxybenzotriazole (HOBt) as an activated ester, *N*,*N*-diisopropylethylamine (DIPEA) as the base, and anhydrous dimethylformamide (DMF) as the solvent, at room temperature for 12 h. This optimized procedure afforded the remaining desired final compounds **3a**,**c**,**f**–**i** with good to optimal yields. ^1^H and ^13^C NMR spectra of the final compounds **3a**–**i** (Figures S1–S9) can be found in the Supplementary Material.

### 3.2. Enzymatic Inhibition Assays

**Figure 4:**
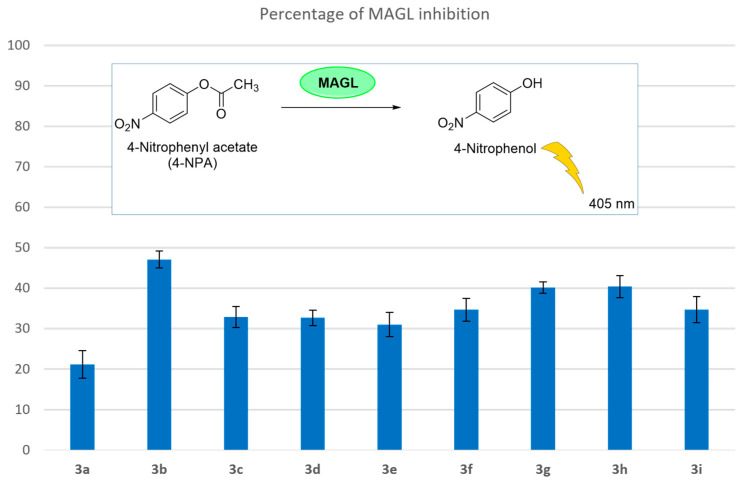
In vitro inhibitory activity (%) on *h*MAGL of derivatives **3a**–**i**.

A spectrophotometric assay, in which 4-nitrophenylacetate was used as the enzyme substrate, was conducted to test the *h*MAGL inhibition activities of the newly synthesized CFAs. The percentages of MAGL enzyme inhibition are reported in Figure 4. The final compounds **3a**–**i** were tested at a single concentration of 50 µM. Among these compounds, **3b** exhibited the highest single-point inhibition percentage (47.1%). For this compound, a full dose–response curve was obtained to calculate its IC_50_ value, which turned out to be 36 ± 4 µM, representing the mean of three independent experiments conducted in duplicate.

## 4. Discussion

It is important to note that all the CFAs tested showed some degree of MAGL inhibition, although there are significant differences to consider. First of all, the series of unsaturated CFAs (**3g**–**i**) generally display higher potencies than most of the saturated derivatives. In particular, compound **3g** that corresponds to CMO, whose anti-arthritis properties had been previously demonstrated in murine models [2], proved to efficiently inhibit MAGL. Similarly, its close analogue **3h** (cetyl palmitoleate), possessing the double bond at the same distance from the ester group as **3g**, proved to be equally active, whereas their longer analogue **3i** (cetyl oleate) displayed slightly weaker potency in the same test, probably due to the extended length of its acyl chain encountering some steric clash with the enzyme active site.

Overall, the series of Δ9-unsaturated fatty acid esters demonstrated that an acyl chain content of 14 to 16 carbon atoms leads to the formation of more potent inhibitors (**3g**,**h**) than 18 carbon atoms (**3i**).

A different behaviour was observed in the saturated series of CFAs, which are generally weaker inhibitors, with the single exception of compound **3b** (cetyl caproate or decanoate), which proved to be the most potent inhibitor of the whole set of CFAs described here. In this case, the optimal length of the acyl chain turned out to be 10 carbon atoms, as shorter (**3a**) or longer saturated acyl groups (**3c**–**f**) produced weaker MAGL inhibitors. Different optimal lengths between the unsaturated vs. saturated derivatives might be ascribed to the distinct conformational freedom of their respective side chains, which may strongly affect their spatial disposition. A full dose–response curve was obtained for the most active inhibitor, **3b**, and its IC_50_ value turned out to be 36 µM, which of course does not represent extremely potent inhibition, especially when compared to other MAGL inhibitors reported in the literature that display activities in the low/sub-nanomolar range. We are aware that these compounds are characterized by weaker potencies when compared to other synthetic MAGL inhibitors reported in the literature [42]. However, a concentration in the micromolar range of **3b** and its close analogues is expected to be readily achieved in musculoskeletal compartments by topical application, thanks to efficient passive absorption through the skin due to the highly lipophilic nature of these compounds. Therefore, we strongly believe that MAGL inhibition levels caused by these CFAs significantly contribute to the overall clinical effectiveness of their preparations. A clear advantage of these CFAs over more potent synthetic MAGL inhibitors is the fact that they are naturally available biomolecules that have been extensively utilized during the past decades in topical formulations to treat musculoskeletal afflictions, and no evident toxicities have so far emerged from their use.

Overall, we think that our findings lay the groundwork for the development and clinical use of small molecules that are able to indirectly activate cannabimimetic pathways to produce a local analgesic and anti-inflammatory effect. This strategy would represent a clear advantage over NSAIDs, which are known to produce gastrointestinal side effects, as well as over opioid analgesic, which instead pose a serious risk of addiction and dependence.

## 5. Conclusions

Taken together, these results provide promising evidence supporting the hypothesis that the analgesic effects of CFAs may be mediated, at least in part, by MAGL inhibition. This potential mechanism offers a novel perspective on the therapeutic applications of CFAs, positioning them as promising candidates for pain management strategies locally targeting the endocannabinoid system. This would not be the first example of the unexpected involvement of cannabinoid mechanisms in widely utilized drugs, such as in the case of a mild analgesic drug, paracetamol [43].

Furthermore, this work may indicate which CFA could be more effective based on this mechanism of action and, therefore, guide companies to optimize the composition of their products. Finally, our results demonstrate that the most potent MAGL inhibitor of this series, cetyl caprate **3b**, which, to the best of our knowledge, is not commonly employed in CFA-based formulations, might potentially induce a more efficient therapeutic outcome and should instead be included in these products. Of course, further investigations are necessary to validate these results, including more in-depth pharmacological evaluations of this class of compounds. Meanwhile, it appears evident that therapeutic interventions producing local cannabimimetic effects may represent novel strategies that can lead to the development of new clinical candidates, with the aim of producing more effective and safer drugs against musculoskeletal afflictions.

## Data Availability

The original contributions presented in this study are included in the article/Supplementary Materials. Further inquiries can be directed to the corresponding author(s).

## Supplementary Materials

The following supporting information can be downloaded at https://www.mdpi.com/article/10.3390/biom15030363/s1, Figure S1: ^1^H-NMR and ^13^C-NMR of compound **3a**; Figure S2: ^1^H-NMR and ^13^C-NMR of compound **3b**; Figure S3: ^1^H-NMR and ^13^C-NMR of compound **3c**; Figure S4: ^1^H-NMR and ^13^C-NMR of compound **3d**; Figure S5: ^1^H-NMR and ^13^C-NMR of compound **3e**; Figure S6: ^1^H-NMR and ^13^C-NMR of compound **3f**; Figure S7: ^1^H-NMR and ^13^C-NMR of compound **3g**; Figure S8: ^1^H-NMR and ^13^C-NMR of compound **3h**; Figure S9: ^1^H-NMR and ^13^C-NMR of compound **3i**.

## Abbreviations

The following abbreviations are used in this manuscript:

CFA	Cetylated Fatty Acid
AEA	Anandamide
2-AG	2-Arachidonoylglycerol
MAGL	Monoacylglycerol Lipase
NSAIDs	Nonsteroidal Anti-Inflammatory Drugs
CMO	Cetyl Myristoleate
ECS	Endocannabinoid System
HESI	Heated Electrospray Ionization
ES-MS	Electrospray Mass Spectrometry
DMSO	Dimethylsulfoxide
