# Supplementary material for: Identification of a Possible Endocannabinoid-Mediated Mechanism of Action of Cetylated Fatty Acids
Source: Biomolecules. 2025 Mar 2;15(3):363. doi: 10.3390/biom15030363 (PMC11940079; doi:10.3390/biom15030363)
Supplement: Supplementary file 1 [file biomolecules-15-00363-s001.zip › biomolecules-3500925-supplementary.pdf]

Supporting Information

Identification of a possible endocannabinoid-mediated mechanism of action of Cetylated Fatty Acids

Giulia Bononi [1,2], Carlotta Granchi [1,2], Tiziano Tuccinardi [1,2] and Filippo Minutolo [1,2*]

[1] Department of Pharmacy, University of Pisa, Via Bonanno 6, 56126 Pisa, Italy.

giulia.bononi@farm.unipi.it (G.B.); carlotta.granchi@unipi.it (C.G.); tiziano.tuccinardi@unipi.it (T.T.)

[2] Center for Instrument Sharing of the University of Pisa (CISUP), Lungarno Pacinotti 43, 56126 Pisa, Italy.

[*] Correspondence: filippo.minutolo@unipi.it (F.M.)

Table of Contents

Figure S1-S9. ^{1}H and ^{13}C NMR spectra of the final compounds **3a-i**. S2-S10

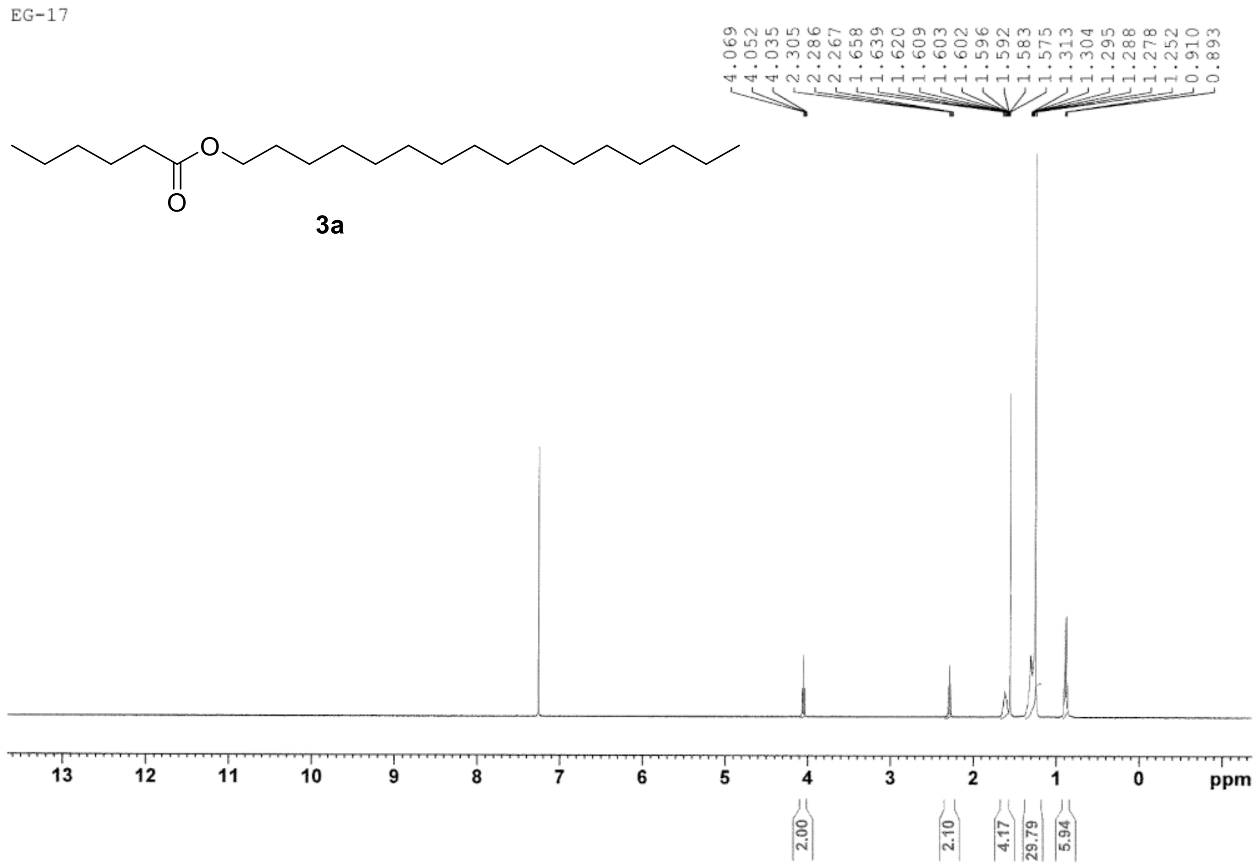

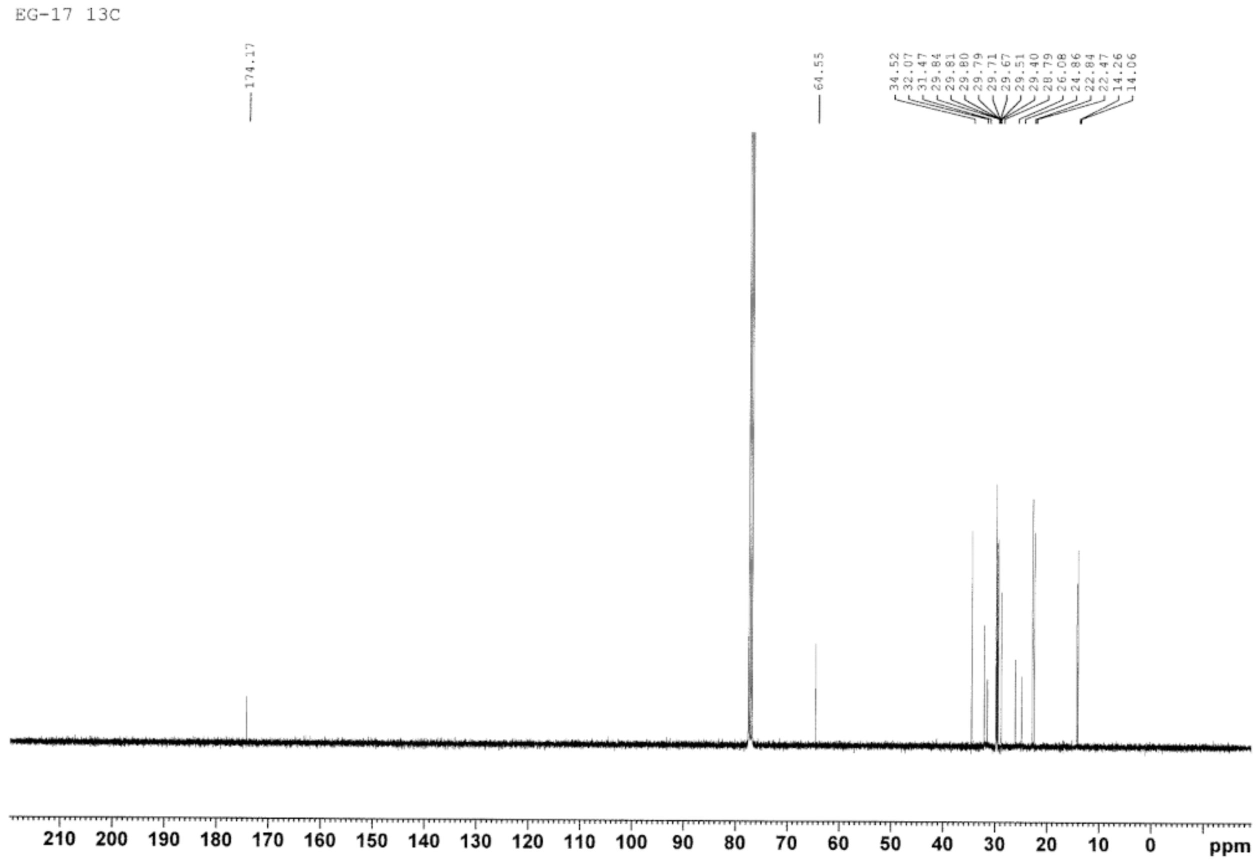

Figure S1. ^{1}H-NMR and ^{13}C-NMR of compound **3a**.

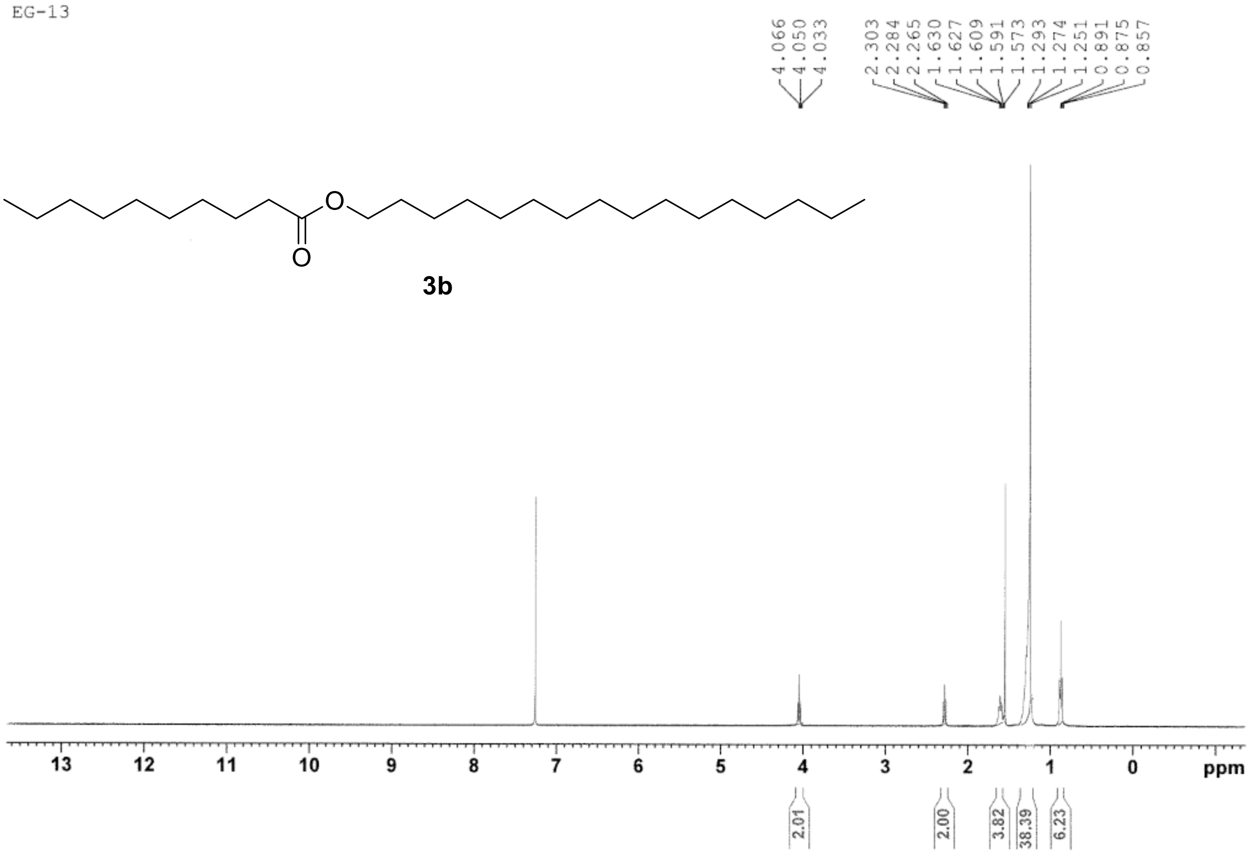

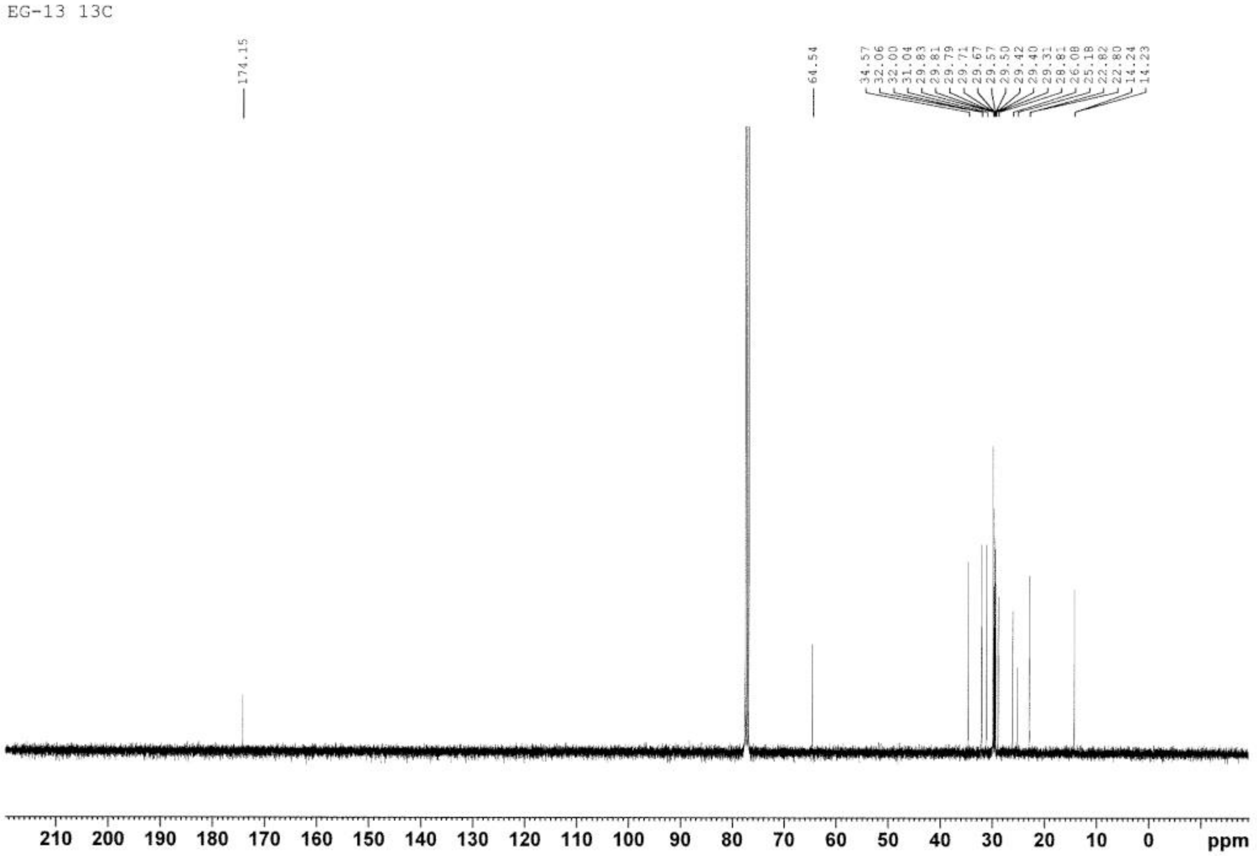

Figure S2. ^{1}H-NMR and ^{13}C-NMR of compound **3b**.

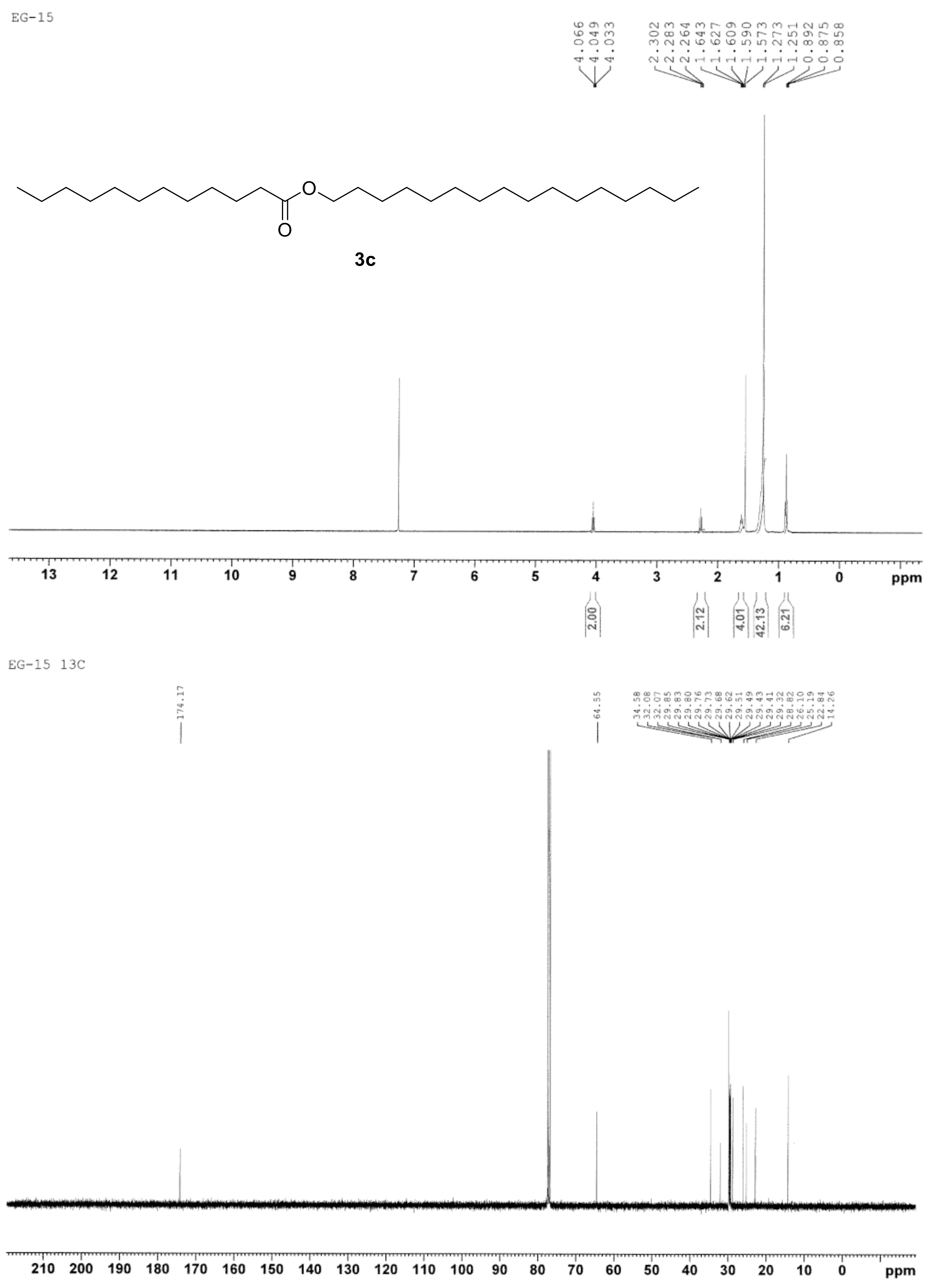

Figure S3. ^{1}H-NMR and ^{13}C-NMR of compound **3c**.

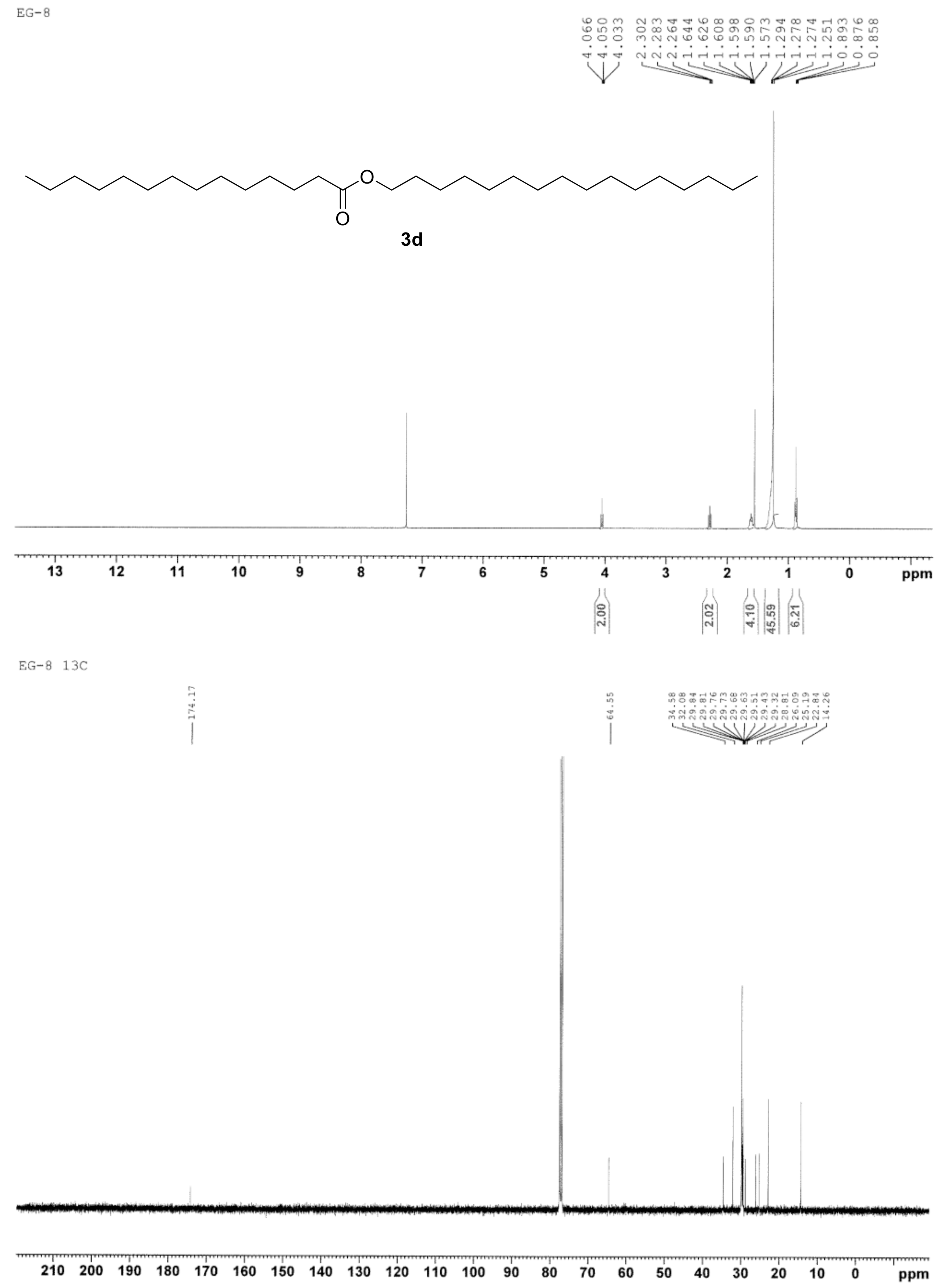

Figure S4. ^{1}H-NMR and ^{13}C-NMR of compound **3d**.

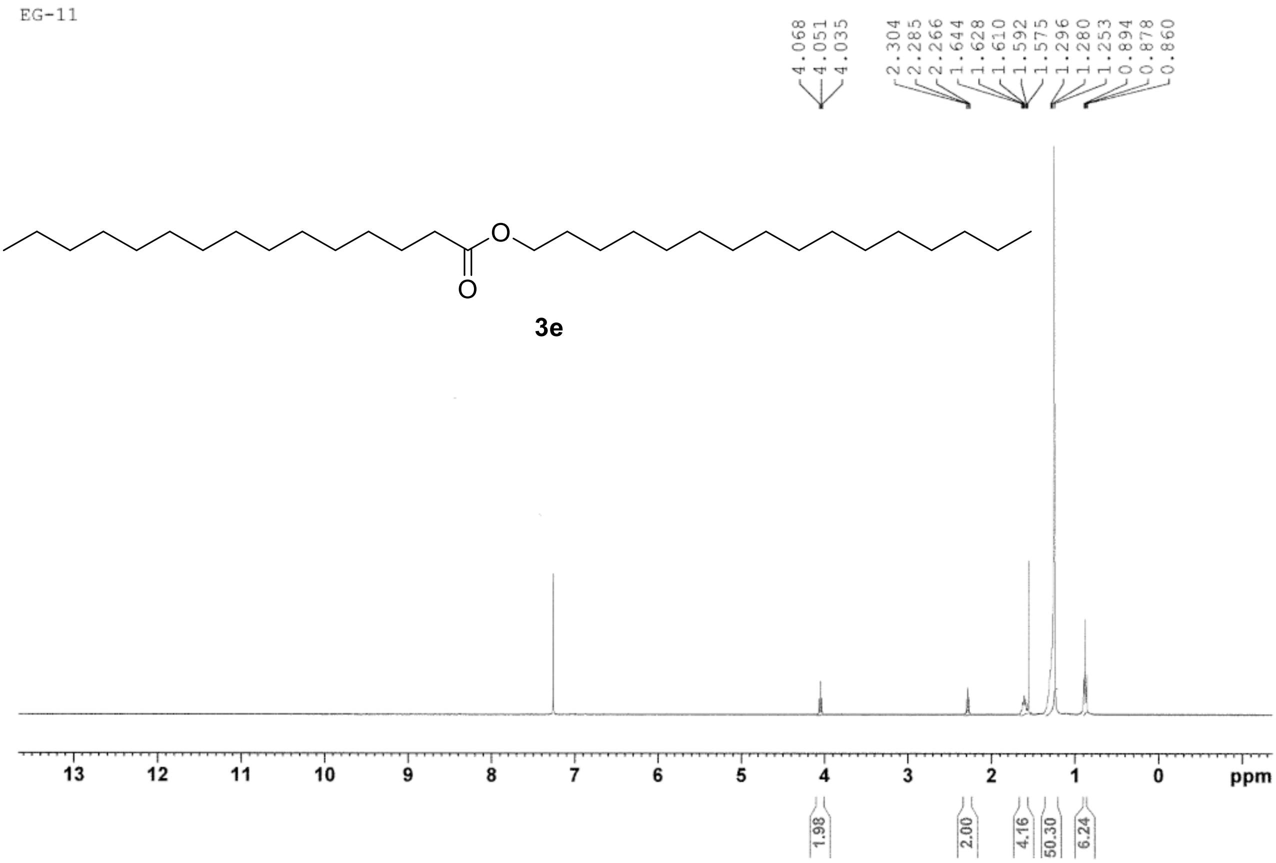

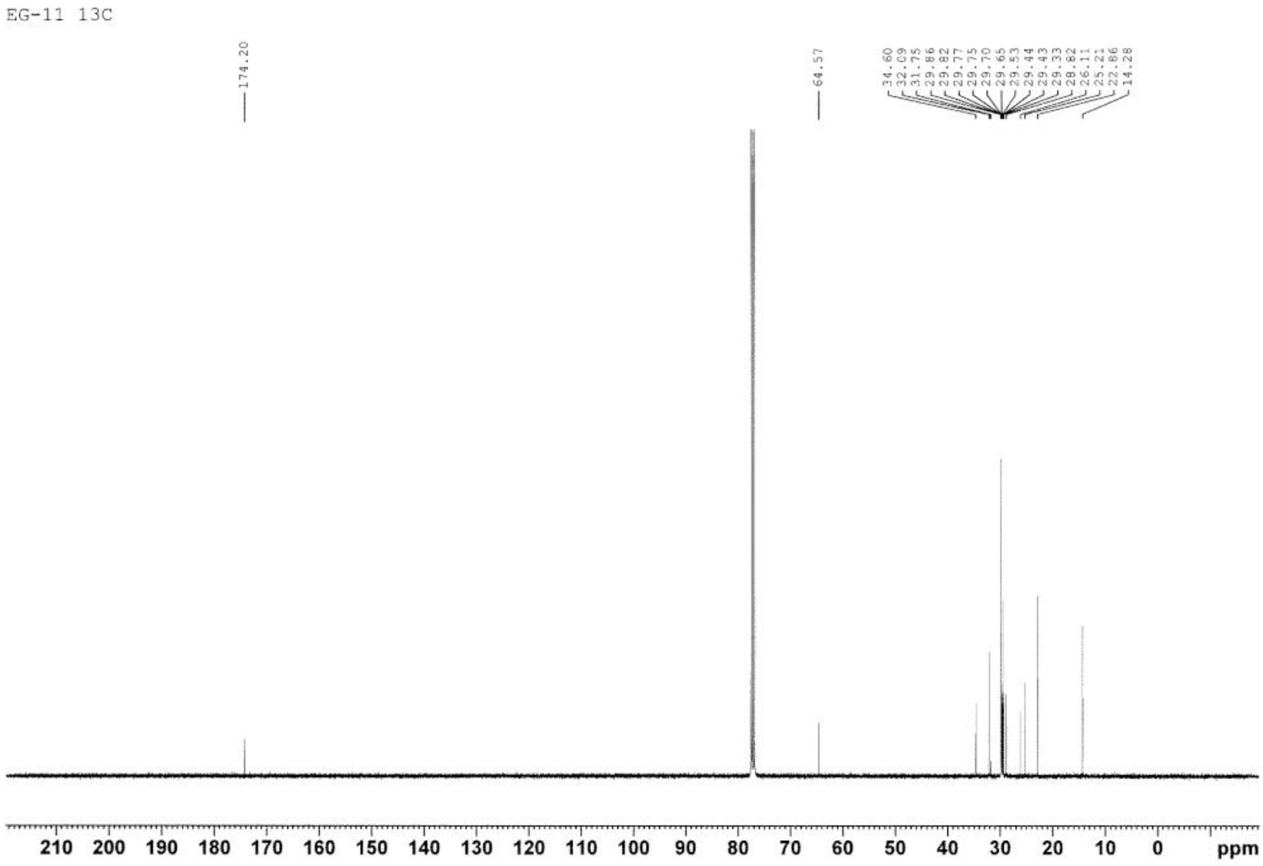

Figure S5. ^{1}H-NMR and ^{13}C-NMR of compound **3e**.

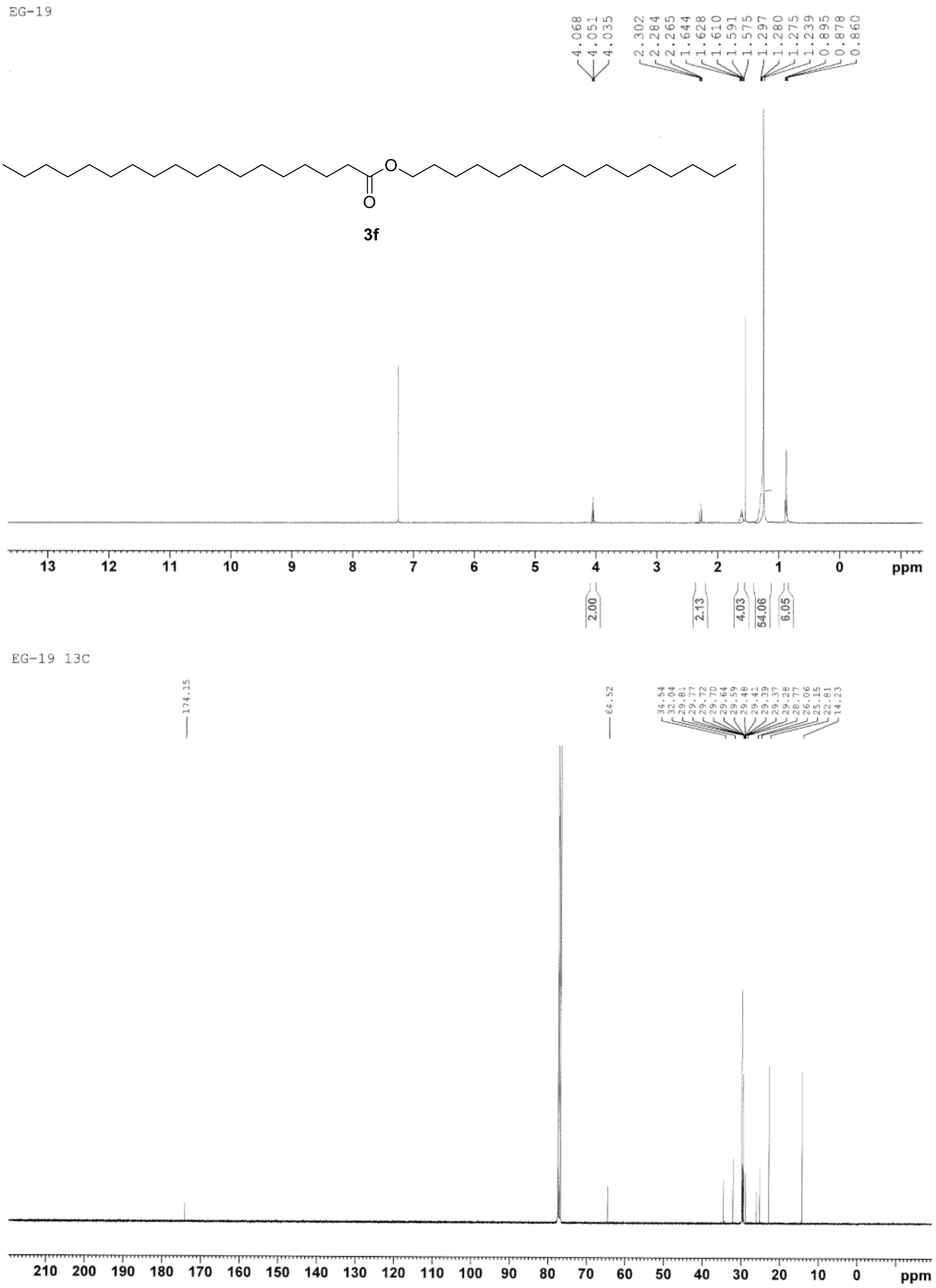

Figure S6. ^{1}H-NMR and ^{13}C-NMR of compound **3f**.

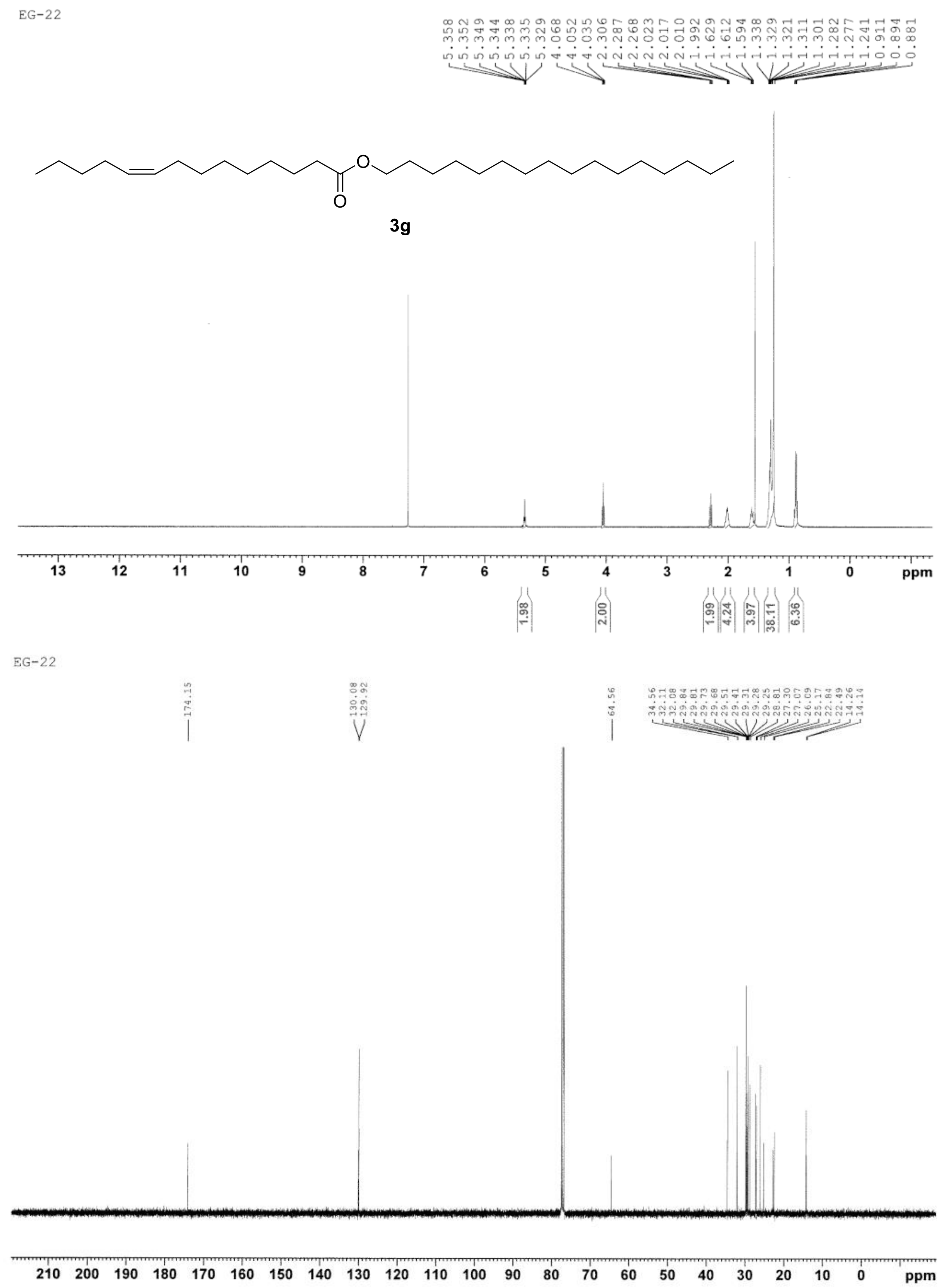

Figure S7. ^{1}H-NMR and ^{13}C-NMR of compound **3g**.

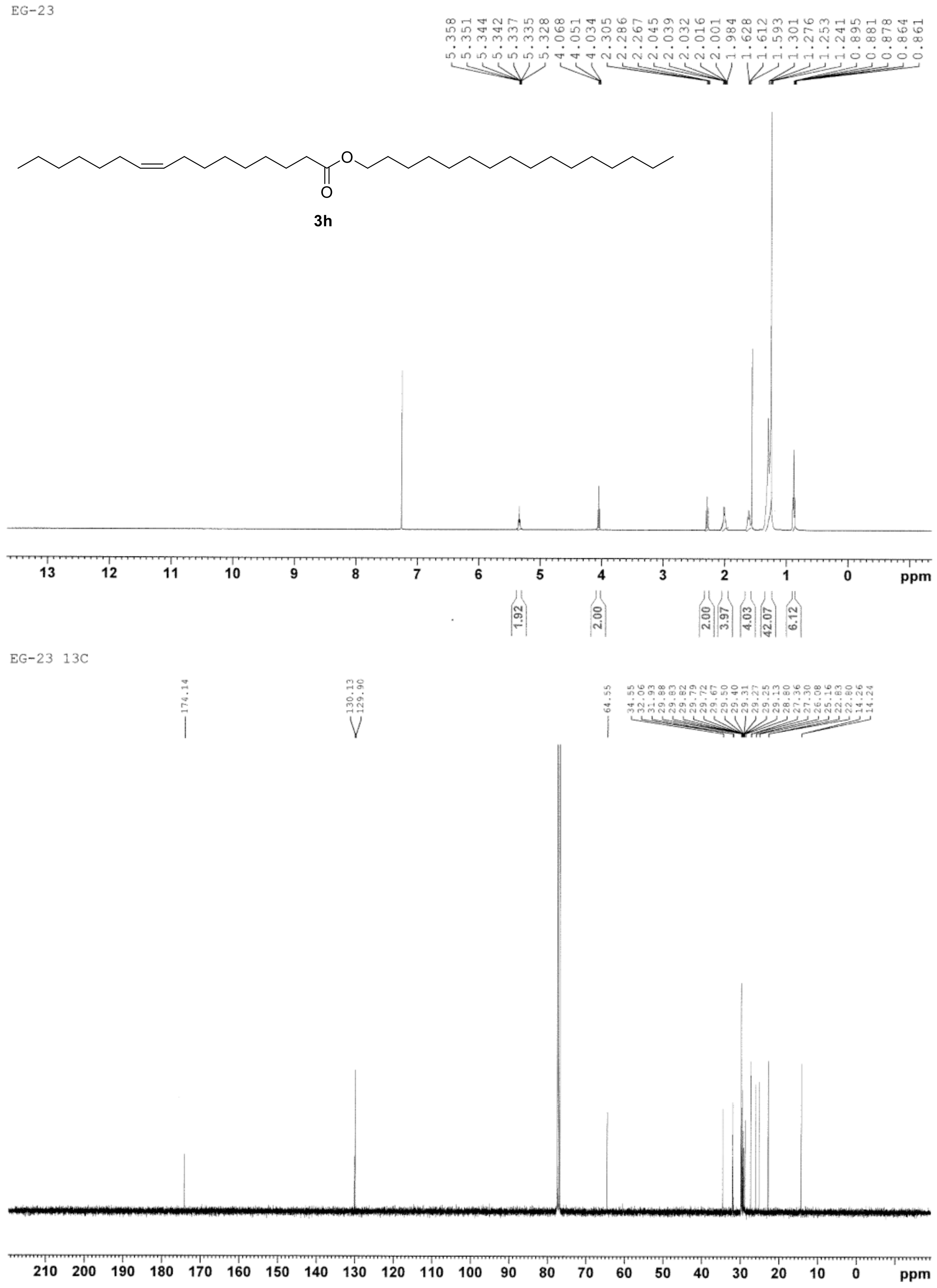

Figure S8. ^{1}H-NMR and ^{13}C-NMR of compound **3h**.

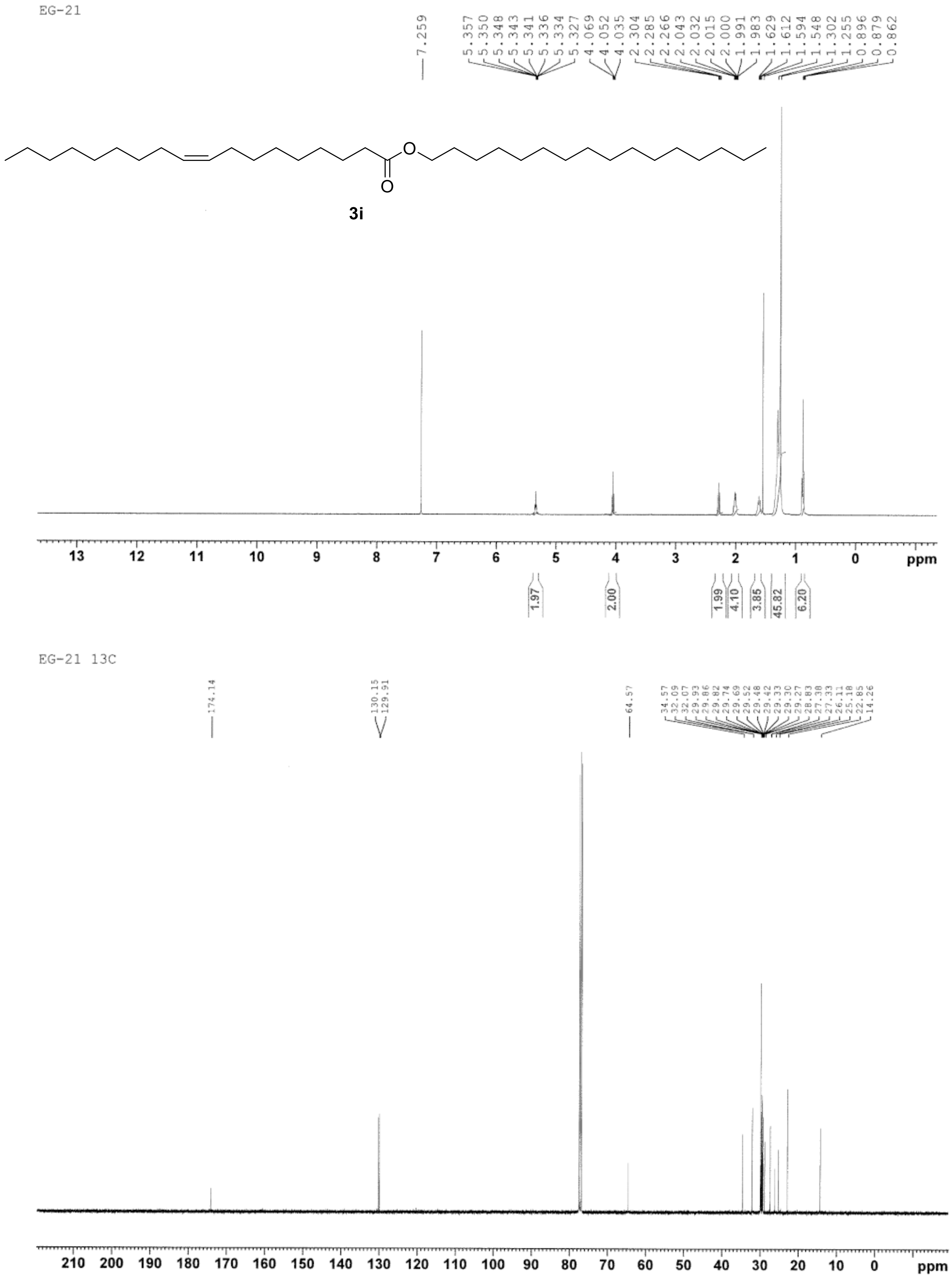

Figure S9. ^{1}H-NMR and ^{13}C-NMR of compound **3i**.
